# The Role of Glia in Canine Degenerative Myelopathy: Relevance to Human Amyotrophic Lateral Sclerosis

**DOI:** 10.1007/s12035-019-1488-3

**Published:** 2019-01-23

**Authors:** Dominika Golubczyk, Izabela Malysz-Cymborska, Lukasz Kalkowski, Miroslaw Janowski, Joan R Coates, Joanna Wojtkiewicz, Wojciech Maksymowicz, Piotr Walczak

**Affiliations:** 1 Department of Neurosurgery, School of Medicine, University of Warmia and Mazury, Olsztyn, Poland; 2 Russell H. Morgan Department of Radiology and Radiological Science, Johns Hopkins University, Baltimore, MD USA; 3 Cellular Imaging Section and Vascular Biology Program, Institute for Cell Engineering, Johns Hopkins University, Baltimore, MD USA; 4 Department of NeuroRepair, Mossakowski Medical Research Centre, Polish Academy of Sciences, Warsaw, Poland; 5 Department of Veterinary Medicine and Surgery, College of Veterinary Medicine, University of Missouri, Columbia, MO USA; 6 Department of Pathophysiology, School of Medicine, University of Warmia and Mazury, Olsztyn, Poland

**Keywords:** Degenerative myelopathy, Demyelination, Disease model, ALS, Monocarboxylate transporters

## Abstract

Amyotrophic lateral sclerosis (ALS) is a fatal neurodegenerative disease characterized by progressive degeneration of motor neurons and grim prognosis. Over the last decade, studies on neurodegenerative diseases pointed on the role of glia in supporting the proper function of neurons. Particularly, oligodendrocytes were shown to be essential through myelin production and supplying axons with energy metabolites via monocarboxylate transporters (MCT). We have used dogs with naturally occurring degenerative myelopathy (DM) which closely resembles features observed in human ALS. We have performed two types of analysis of spinal cord tissue samples: histology and molecular analysis. Histology included samples collected from dogs that succumbed to the DM at different disease stages, which were compared to age-matched controls as well as put in the context of young spinal cords. Molecular analysis was performed on spinal cords with advanced DM and age-matched samples and included real-time PCR analysis of selected gene products related to the function of neurons, oligodendrocytes, myelin, and MCT. Demyelination has been detected in dogs with DM through loss of eriochrome staining and decreased expression of genes related to myelin including MBP, Olig1, and Olig2. The prominent reduction of MCT1 and MCT2 and increased MCT4 expression is indicative of disturbed energy supply to neurons. While Rbfox3 expression was not altered, the ChAT production was negatively affected. DM in dogs reproduces main features of human ALS including loss of motor neurons, dysregulation of energy supply to neurons, and loss of myelin, and as such is an ideal model system for highly translational studies on therapeutic approaches for ALS.

## Introduction

Amyotrophic lateral sclerosis (ALS) is a neurodegenerative disorder with an inevitable progression, extremely poor prognosis, and no cure till now. While ALS is relatively rare, its occurrence increases with age, so the burden of this deadly disease will be growing, as societies age [1]. In the USA, there are approximately 5600 people diagnosed with ALS every year. ALS affects both upper and lower motor neurons (UMN, LMN). Progressive damage of these neurons leads to lack of muscle stimulation and atrophy. The involvement of respiratory muscles usually leads to death [2]. Historically, ALS has been considered as a disorder of motor neurons, and accordingly, a majority of research efforts focused on this aspect. Currently, there is a growing appreciation for the role of the microenvironment and all neural phenotypes in the brain [3]. The studies conducted during the last decade have raised the supportive role of glia in the homeostasis and survival of neurons. Oligodendrocytes, due to their unique spatial arrangement and large contact surface with motor neurons, seem to be extremely important. Oligodendrocytes are well known for their role in producing myelin, which ensheaths and insulates axons in the central nervous system (CNS), facilitating rapid and energy-efficient conduction of action potentials. Recent studies have demonstrated that the role of oligodendrocytes extends beyond myelination in the maintenance and long-term survival of neurons [4]. The energy demands of firing neurons are supplied along the entire length of axons, which, in the case of motor neurons, spans up to 1 m. Condensed insulation of the axon by the myelin membrane, except for the nodes of Ranvier, creates a significant barrier for energy substrate transfer to the axon, which precipitates the need for a mechanism to facilitate local feeding of axons by oligodendrocytes. As shown by Lee and others [4], oligodendroglia do, indeed, participate in the transportation of energy substrates into axons through specialized monocarboxylate transporters (MCT).

Extensive activation of the most abundant glial component—astrocytes—was seen both in ALS patients and in dogs with degenerative myelopathy (DM) [5, 6]. Astrocytes are significant contributors to neuronal dysfunction and death in ALS pathology. Moreover, it was reported that co-culture of astrocytes from ALS patients, both familial and spontaneous cases, is toxic and leads to the death of motor neurons [7]. Furthermore, astrocytes isolated from sporadic ALS (sALS) patients and transplanted to a mouse model resulted in degeneration of the motor neurons and non-motor neurons of the host [8]. These observations about non-cell autonomous pathology are largely derived from transgenic animal models, which are clearly useful tools; however, there is an increasing criticism that artificially created animal models of human disease are not reliable for the evaluation of treatment outcomes, as they do not address the disease pathophysiology and variability existing in human population. Canine DM is an inherited, progressive, adult-onset neurodegenerative disease with clinical, histopathologic, and genetic parallels to human ALS, including presence of mutated superoxide dismutase 1 gene (*SOD1*) [9]. The similarities between the canine and human nervous systems, exposition to similar environmental factors as human, the homogeneity in the onset and clinical progression of disease, and the ability to longitudinally analyze and collect samples from affected dogs with DM represent a uniquely valuable large animal disease model for therapeutic development.

**Fig. 1:**
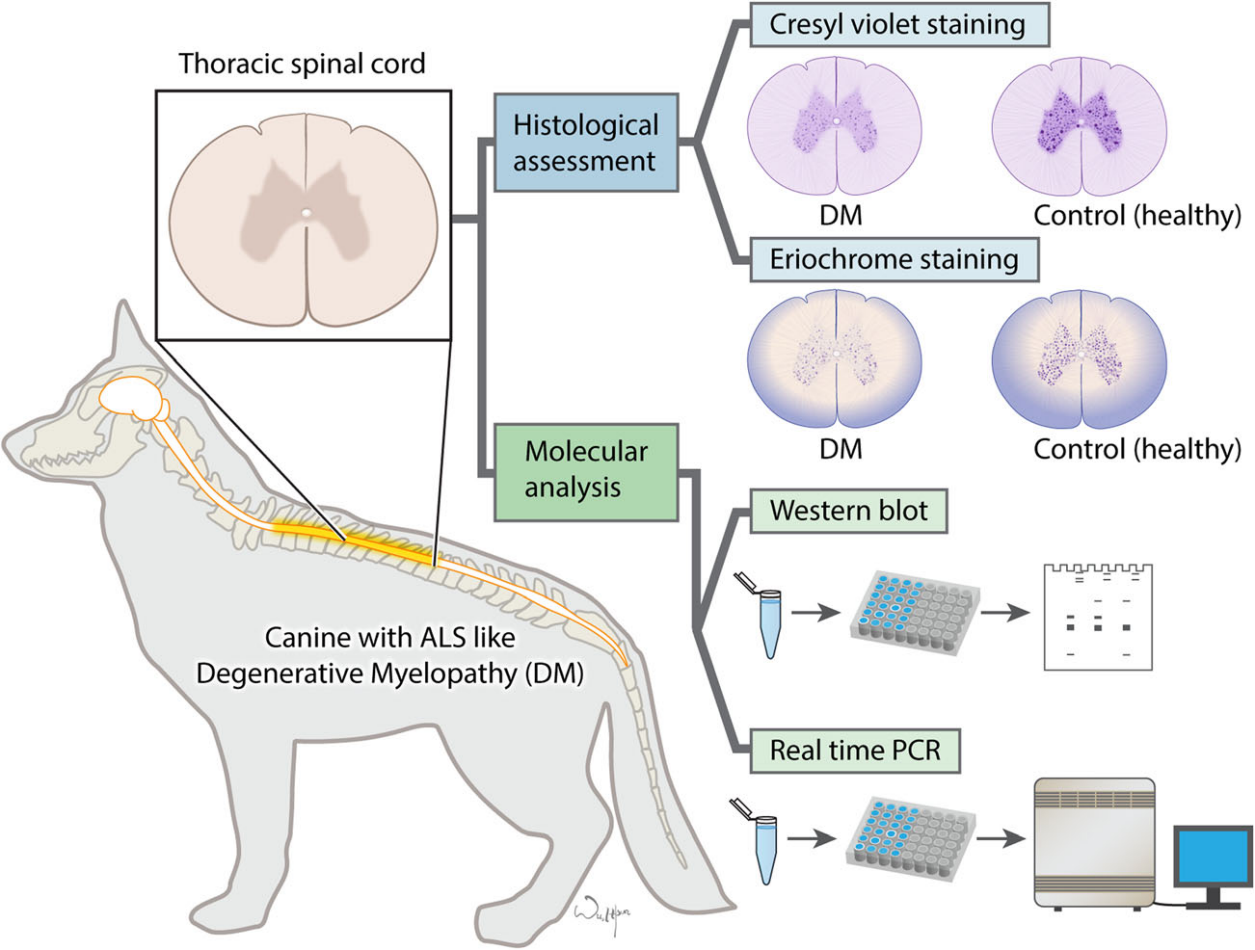
Experimental design

Our objective was to evaluate whether glia, and their MCTs may play a significant role in the degeneration of motor neurons in canine DM similarly to previously reported for transgenic *SOD1* mice [4]. This study was focused on the assessment of the status of the glial component, including mature astrocytes, and myelinating oligodendrocytes, as well as MCT in the spinal cord to give a basis for using DM to investigate glia-focused therapeutic strategies in a highly translational setting of large animals with ALS-like disease (experimental design is shown in the Fig. 1).

## Results

### Quantitative Analysis of Motor Neurons

**Fig. 2:**
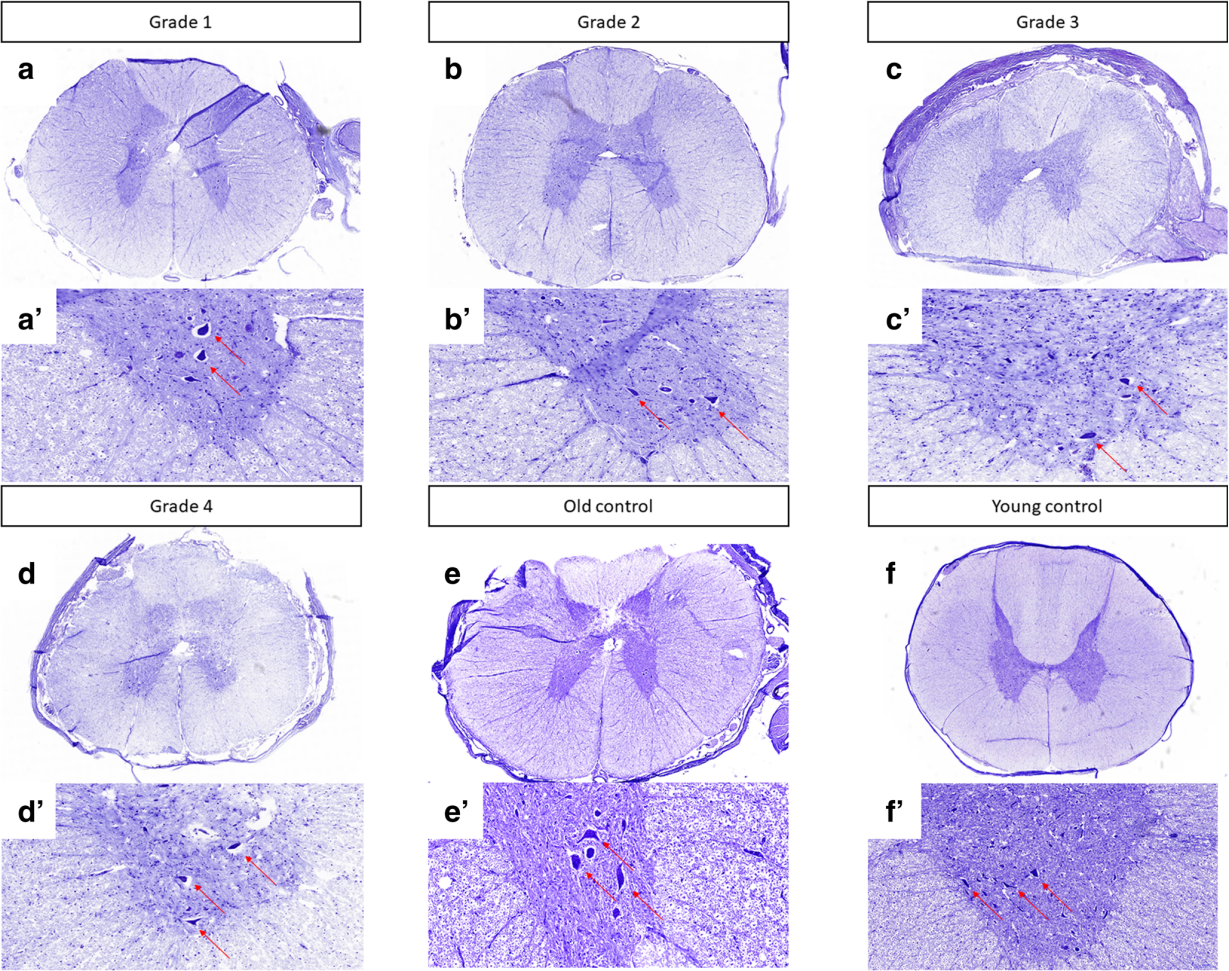
Cresyl violet staining of canine spinal cords. (A, A’) Grade 1. (B, B’) Grade 2. (C, C’) Grade 3. (D, D’) Grade 4. (E, E’) Old control dogs. (F, F’) Young control dogs. Letters with apostrophe correspond to pictures marked with capital letters, × 10 magnification

Cresyl violet staining revealed characteristic large-sized motor neurons within the spinal cord (Fig. 2). We performed motor neuron counts in both old and young controls and did not observe significant differences between those two groups, thus excluding effect of the age. Quantitative analysis has shown that the number of motor neurons was reduced in dogs with DM, compared to both young and old control (unaffected) animals. The highest reduction of motor neurons was seen in most advanced, grade four DM (12.00 ± 0.84 in DM-affected animals vs. 37.10 ± 1.67 in old control animals). There was clear progression of the motor neuron loss with progression of the disease, and starting from DM grade 2, the difference was statistically significant when compared to age-matched control groups (*p* < 0.05). Moreover, we evaluated a number of motor neurons in the region of lamina IX, which contained somatic neurons. We were unable to determine any correlations between grade and number of motor neurons in this region. The highest reduction in number of motor neurons was in dogs with grade four of DM (9.412 ± 0.91).

### The Assessment of Demyelination

**Fig. 3:**
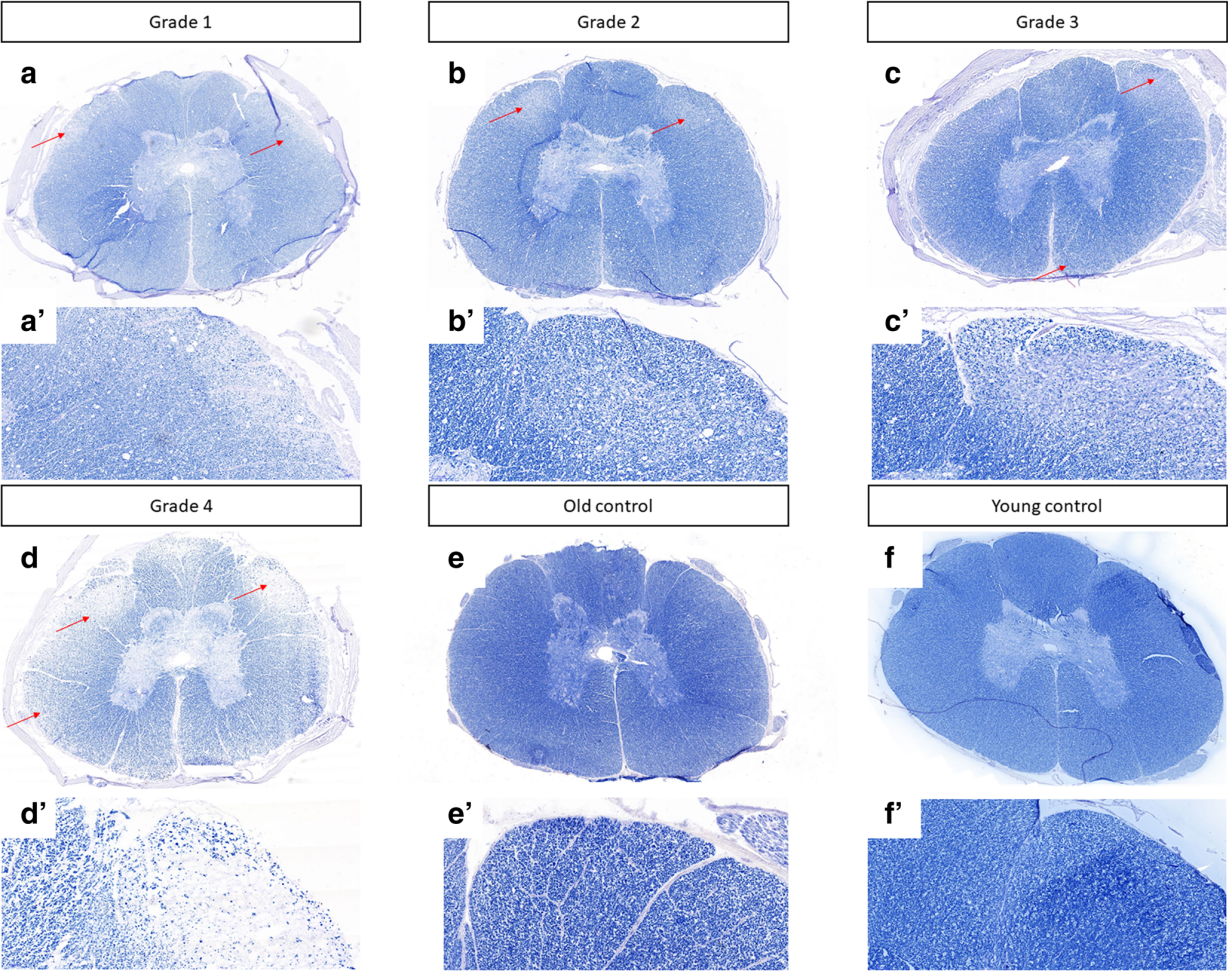
Eriochrome staining of canine spinal cords. (A, A’) Grade 1. (B, B’) Grade 2. (C, C’) Grade 3. (D, D’) Grade 4. (E, E’) Old control dogs. (F, F’) Young control dogs. Letters with apostrophe correspond to pictures marked with capital letters, × 10 magnification; arrows indicate loss of blue staining in the peripheries of tissue

**Fig. 4:**
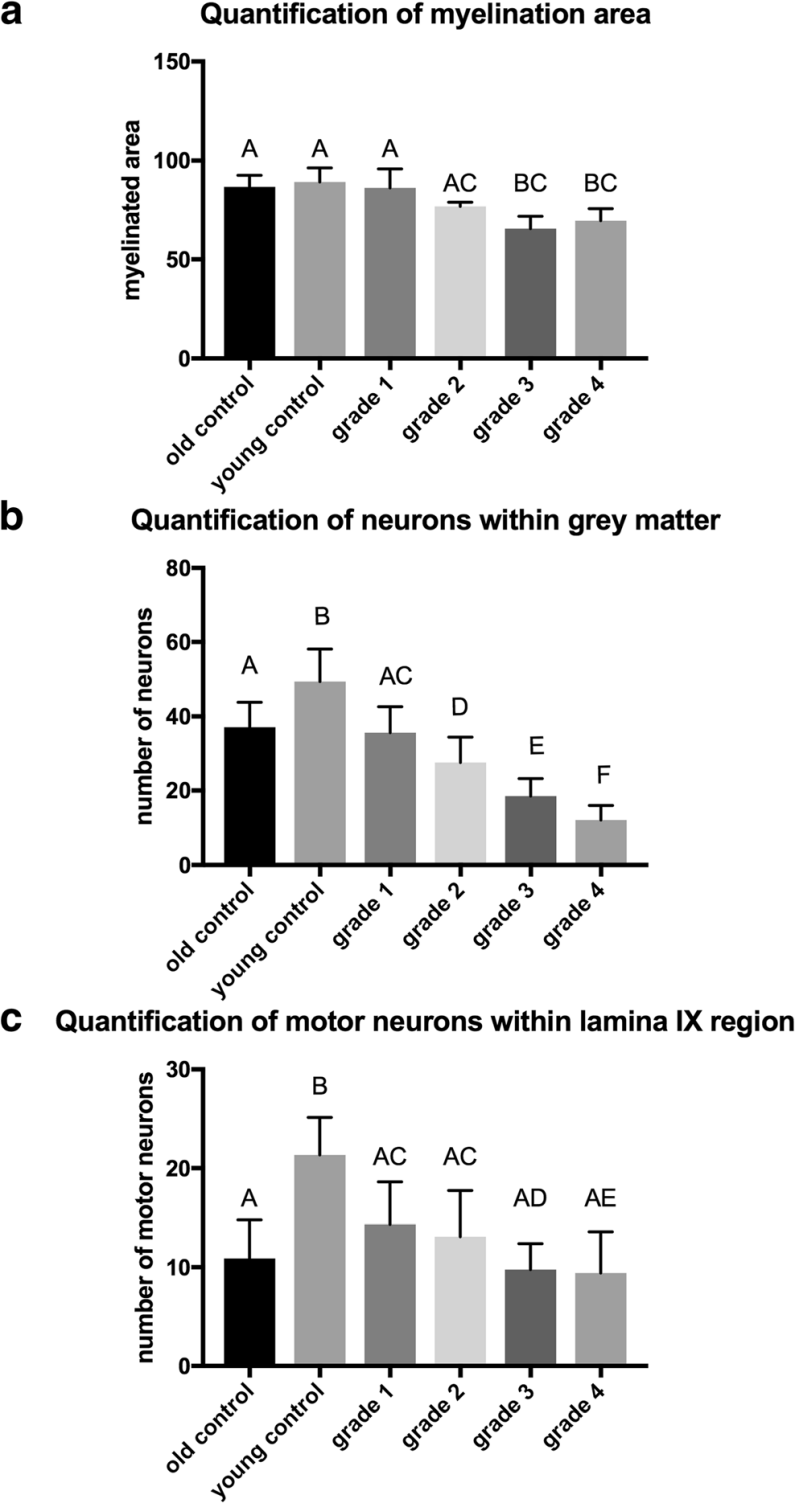
Quantification of eriochrome (**a**) and Cresyl violet (**b**, **c**) staining

Eriochrome staining enabled myelin visualization in the spinal cord. The dark blue color of eriochrome was prominent throughout white matter in control dogs. Regions of demyelination were seen as voids of the blue color primarily on the outer surface of the spinal cord, as shown in Fig. 3, in dogs with initial stages of disease. Whereas, the brightening of blue color was evident in dogs with advanced DM. Results are shown in Fig. 4. There was a significant decrease in the myelination level of the tissues of DM dogs compared to controls. There was a significant difference starting from grade 3 (65.57 ± 2.56) compared to old control dogs (86.69 ± 2.92).

### Molecular Analysis of Matched Spinal Cord of Dogs Affected with DM

The expression of different genes in two groups (the unaffected—control and DM-affected) of spinal cords was measured. The DM-affected dogs had a higher expression of genes related to microglia, including AIF1 (*p* < 0.0001) and lower expression of genes related to microglia including *MBP* (*p* = 0.02), *Olig1* (*p* = 0.025), and *Olig2* (*p* = 0.048). There was no significant difference in the expression of *GFAP*, *MAG*, or the *MOG* gene (*p* > 0.05), but we observed a downward trend in the expression profiles of the *MAG* gene.

**Fig. 5:**
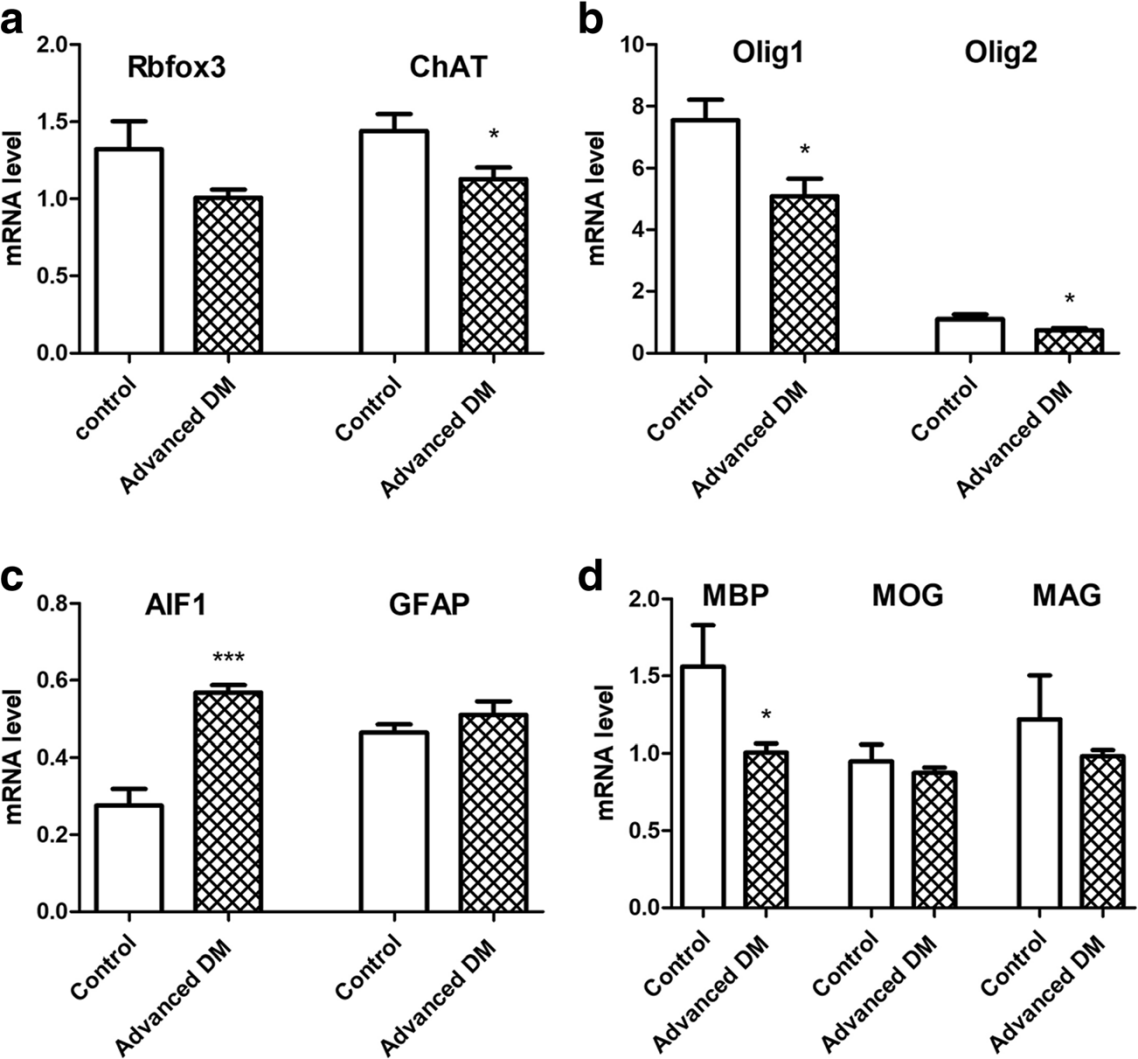
Real-time PCR analysis. (**a)** Genes related with neurons. (**b)** Genes related with oligodendrocytes. (**c)** Genes related with microglia (*AIF1*), and astrocytes (*GFAP*). (**d**) Genes related with myelin

Disease did not influence *Rbfox3* expression (Fig. 5a), but it markedly affected the expression of the *ChAT* gene (Fig. 5a), which encodes an enzyme that catalyzes the biosynthesis of the neurotransmitter acetylcholine.

**Fig. 6:**
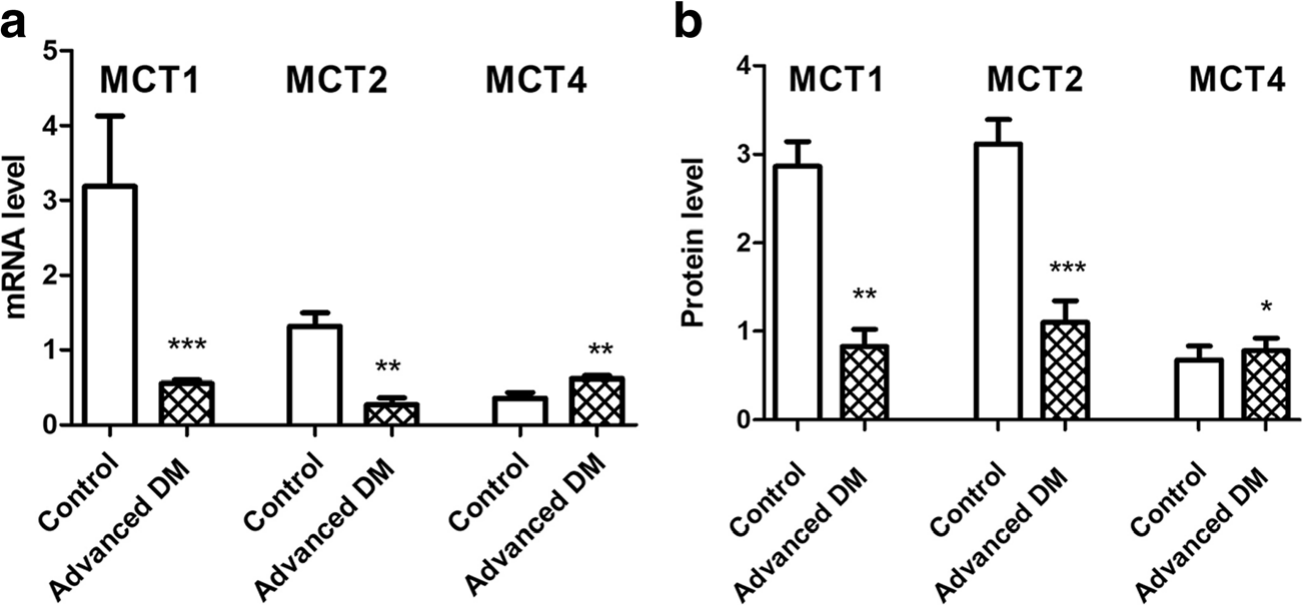
Molecular analysis of analysis of MCT (monocarboxylate transporters). (**a**) Real-time PCR and (**b**) Western blot analysis

There were significant differences in the expression of *MCT1* (*p* = 0.0005; Fig. 6a), *MCT2* (*p* = 0.0012; Fig. 6a), *MCT4* (*p* = 0.0075; Fig. 6a) responsible for coding a proton-linked monocarboxylate transporter that catalyzes the movement of many monocarboxylates, such as lactate and pyruvate, across the plasma membrane. Western blot analysis of proteins MCT1, -2, and -4 showed a prominent reduction in MCT1 and -2 proteins in samples from DM-affected dogs (Fig. 6b). We noticed an increase in the level of the MCT4 protein level compared to unaffected dogs.

## Discussion

Over the last decade, multiple transgenic animal models of oligodendroglia injury have been investigated. All of these have demonstrated axonal pathology independently. This may suggest that myelin pathology causes axonal injury in ALS. This led us to evaluate the glia component in dogs that are affected by DM. Previous papers have outlined the histopathological changes in the spinal cords of dogs affected by DM [10–13]. Prior histopathologic studies of canine DM showed a complete loss of axon/myelin cylinders replaced by prominent astrogliosis [6, 12]. Our results confirmed demyelination and neuronal degeneration in the spinal cords of dogs affected by DM. Regions of demyelination were seen as the absence of blue EC staining (Fig. 3). Loss of blue staining was most prominent in the spinal cord of a dog with grade four DM. In spinal cords affected by DM, the lack of myelin was most noticeable in the periphery of the spinal cord, which corresponds to the location of the dorsal spinocerebellar pathways and is in line with the observations by March et al., 2009 [12]. Significant differences were present between grade three and four compared to control old unaffected dogs. No significant loss of myelin was observed in the spinal cords of the control dogs, which led us to hypothesis that such loss of myelin could be related to the grade of DM, and occurs in a correlation with axonal degeneration and secondary demyelination as shown with cresyl violet staining (Fig. 4). Along with the loss of myelin with the grade of DM, we also observed a loss of neurons in the spinal cord, even in grade 2 (Fig. 2). The reduction of neuron numbers in the gray matter in each grade of DM was statistically significant compared to controls [14]. ChAT gene expression was reduced corresponding to histological analysis. Moreover, ChAT activity was markedly reduced in analyzed spinal cords from ALS patients which confirms clinical relevance of our model [15, 16]. Loss of myelin ensheathing in the spinal cord was confirmed with quantitative analyses based on the expression of genes *MBP* (myelin basic protein), *Olig1*, and *Olig2*. The examination of MCT provided a great opportunity to investigate whether primary myelin degeneration has any impact on the degeneration of motor neurons. A lower mRNA expression of *MCT1* was found in a mouse model of ALS resulting in neuron cell loss, reported by Lee et al., in 2012. Similarly, we found a decrease in the both mRNA expression and protein level of MCT1 within a group of dogs with advanced DM. This confirms the crucial role of those transporters in the course of DM as well as the value of DM as a naturally occurring disease model of ALS. *MCT1* is expressed predominantly in oligodendrocytes, *MCT2* in neurons, and *MCT4* in astrocytes [17]. An increase in the *MCT4* which is expressed predominantly in astrocytes can be correlated with an increase in the number of both astrocytes and microglia within the spinal cord, which was previously shown by other investigators [6, 11, 18]. We confirmed statistically significant increases of microglia by *AIF1* gene expression within spinal cord from dogs with DM, but we were not able to see significant increase of expression of *GFAP* gene in those samples. However, we have observed an increasing trend in expression of *GFAP* gene. A decrease in *MCT2* presumably can be linked with a decrease in the expression of the *Olig1* and *Olig2* genes. We hypothesized that degeneration of oligodendrocytes leading to poor myelination of axons was connected to neuronal degeneration and lower expression of *MCT2*. Further investigation should be focused on expression of these genes on different stages of disease. Our study supports the thesis that glial component as well as MCT are affected in this naturally occurring ALS-like disease in dogs. Evidence about the role of glia in DM is mounting [19], and it is essential to undertake further studies on glia replacement. While transplanted glial progenitors were shown to be highly efficacious in dysmyelinated mice [20], translation of this approach to humans largely failed [21]. Extreme differences in brain size between rodents and human make clinical translation challenging, and in this context, it is essential to perform future studies in more clinically relevant disease models such as dogs affected by DM [22].

## Materials and Methods

### Sample Collection

We have received tissue samples courtesy of JRC (Dept. Veterinary Medicine and Surgery, College of Veterinary Medicine, University of Missouri, USA). Mid to lower thoracic spinal cord segments were collected immediately after euthanasia and frozen for total RNA and protein extraction, or fixed in 4% formalin for histological examination. This region of the spinal cord was selected because the histopathologic changes become apparent early in the disease process and progress in severity throughout the disease course [12]. Tissues were obtained from dogs euthanized due to a diagnosis of presumptive DM, or from older dogs (control) euthanized from other non-neurologic disease causes. Young (< 5 years) and older (> 9 years) dogs were included to evaluate for the effect of age on the number and condition of motor neurons in histological assessment. Control dogs were euthanized due to unrelated diseases (e.g., blood in stool, weight loss, orthopedic diseases, or chronic renal disease, or abdominal mass; or were euthanized as a part of another study as control animal). Samples from DM-affected dogs were included if the following criteria were met: (1) clinical history and neurologic examination consistent with DM, with assignment to disease stage based on a clinical grading scale (Table 1) [9]; (2) homozygosity for 118G > A *SOD1* mutation; and (3) histopathologic diagnosis of DM that confirmed axonal degeneration and astroglial proliferation that was most severe in the dorsal portion of the lateral funiculus and in the dorsal funiculi of the caudal thoracic spinal cord. The severity of DM was rated according to a grading scheme [9]. Initially, clinical signs emerge as an asymmetric spastic paraparesis and general proprioceptive (GP) ataxia (stage I), progressing to non-ambulatory paraparesis/paraplegia (stage II) with the emergence of pelvic limb lower motor neuron signs within 1 year of disease onset. Dog owners often elect euthanasia when their dog becomes non-ambulatory in the pelvic limbs. Neurologic deficits progress to include thoracic limb weakness (stage III) followed by flaccid tetraplegia, generalized muscle atrophy, and brainstem dysfunction with eventual respiratory failure (stage IV). In total, 17 dogs were included in histological assessment (12 DM dogs, 2 age-matched controls, 3 young control animals), and 9 dogs were included in molecular analysis (5 DM dogs and 4 age-matched controls). The number of animals used in the experiments is shown in Table 1. Outlined experimental design is shown in the Fig. 1.

**Table 1:** Tissues used in experiments

	Histological assessment						Molecular Analysis	
	Grade 1	Grade 2	Grade 3	Grade 4	Young control	Old control	DM-affected dogs (grades 3 and 4)	Control
Number of dogs	*n* = 3	*n* = 3	*n* = 3	*n* = 3	*n* = 3	*n* = 2	*n* = 5	*n* = 4
Age (years)	11.45 ± 1.36	10.33 ± 2.87	13 ± 1.35	12.6 ± 0.66	1	13.35 ± 1.13	12.59 ± 1.84	9.3 ± 4.1
Breed	Boxer (2), Chesapeake Bay Retriever	Boxer, Australian Shepherd mix, Pembroke Welsh Corgi	Pembroke Welsh Corgi (3)	Alaskan Malamute mix, Pembroke Welsh Corgi (2)	Beagle	Pembroke Welsh Corgi (2), Chesapeake Bay Retriever, Rhodesian Ridgeback	Bernese Mountain dog, Pembroke Welsh Corgi (3), Boxer (2), Chesapeake Bay Retriever	Rhodesian Ridgeback (2), mixed breed (2)
Sex	2 F, 1 M	2 F, 1 M	3 F	2 M, 1 F	3 F	4 M	3 F, 2 M	4 F

### Histological Assessment

Tissue samples protected in 4% formalin were cryoprotected in 30% sucrose and then frozen on dry ice. Next, samples were cryosectioned on a Hyrax C25 PLMC cryostat (Zeiss, Warsaw, Poland) in 10-μm sections, mounted on slides, and stored at − 20 °C.

#### Eriochrome Staining

For eriochrome (EC) staining, sections were rehydrated in a graded series of ethanol (ETOH; 95%, followed by 70% ETOH and distilled water), 10 min each at room temperature. Next, sections were stained for 15 min using an eriochrome cyanine solution. Subsequently, sections were differentiated by alternating between exposure to 0.1% NH_4_OH for 3–7 s and rinsing in distilled water for ~ 30 s until the blue background was reduced. The total time of exposure to NH_4_OH was approximately 20 s. After the last rinse in distilled water, sections were dehydrated in 70%, 95%, and 100% alcohol (two times for each), overexposed in xylene (three times for 10 min), and mounted using DPX mounting medium (Sigma-Aldrich, USA). Myelin stained with eriochrome cyanine R was visualized and assessed using bright-field microscopy. Images were captured under × 20 magnification using an Axio Scan.Z1 (Zeiss) and the myelination areas were analyzed using Adobe Photoshop CC software (San Jose, CA, USA). Areas of the spinal cord were outlined using the magnetic lasso tool; the pictures were converted to grayscale, and then measured. The myelination area was measured with the threshold at the level of 148 to analyze the percent of myelination.

#### Cresyl Violet Staining

For cresyl violet staining, sections were rehydrated in a graded series of ethanol dilutions (ETOH; 95%, followed by 70% ETOH and distilled water), for 3 min each at room temperature. Then, sections were rinsed in distilled water and transferred for one and 0.5 min to cresyl violet solution heated to 60 °C. Sections were dehydrated in 70%, 95%, and 100% alcohol, overexposed in xylene (two times for 10 min), and mounted using DPX mounting medium (Sigma-Aldrich, USA). Neurons stained with cresyl violet were visualized and assessed using bright-field microscopy. Images were captured under × 20 magnification using an Axio Scan.Z1 (Zeiss), and the number of neurons is analyzed using ZEN software (Zeiss). For the statistical analysis, the total number of neurons within gray matter and motor neurons within Rexed lamina IX were used and compared between animals.

### Real-time PCR

#### RNA Extraction and Reverse Transcription

Total RNA was extracted from spinal cord samples using Total RNA Plus concentrator according to the manufacturer’s protocol (A&A Biotechnology, Poland). The RNA purity and concentration were measured using a NanoDrop 1000 spectrophotometer (Thermo Fisher Scientific Inc., USA). All sample purity ratio of 260 nm and 280 nm was between 1.9 and 2.0. The next step was reverse transcription of RNA to cDNA using a High-Capacity RNA-to-cDNA™ Kit (Thermo Fisher Scientific Inc., USA). Two types of controls were used: one without complementary DNA (cDNA), and the second, in the absence of the reverse transcriptase. The obtained cDNA was diluted in nuclease-free water and stored at − 80 °C until analysis.

#### Real-time PCR

Real-time quantitative PCR was achieved using Applied Biosystems 7900HT Fast Real-time PCR System (Applied Biosystems, USA). PCR conditions included the following: an initial denaturation step (10 min at 95 °C), followed by 45 cycles of denaturation (15 s at 95 °C), and annealing (60 s at 60 °C). All samples for each gene consisted of the following: 3.5 μl of the sample, 5 μl of the TaqMan Real-time PCR Master Mix (Thermo Fisher Scientific Inc., USA), and 0.5 μl of the TaqMan assay (Table 2, Thermo Fisher Scientific Inc., USA) filled to 10 μl with nuclease-free water. Expression levels were normalized to GAPDH, and relative expression was calculated using the comparative threshold cycle method (∆CT).

**Table 2:** TaqMan assays used in real-time PCR experiment

No.	Gene name	Identification number	No.	Gene name	Identification number
1.	*MBP*	Cf02641118_m1	8.	*MCT4*	Cf02702665_g1
2.	*GFAP*	Cf02655693_g1	9.	*ChaT*	Cf02724445_m1
3.	*AIF1*	Cf02653363_m1	10.	*MOG*	Cf02651312_m1
4.	*Olig1*	Cf02685151_s1	11.	*MAG*	Cf02637655_m1
5.	*Olig2*	Made to order	12.	*Rbfox3*	Cf02658562_m1
6.	*SLC16A1*	Cf02629517_m1	13.	*GAPDH*	Cf04419463_gH
7.	*MCT2*	Made to order	14.	*PPIH*	Cf02665149_m1

### Western Blotting

Frozen spinal cord samples were homogenized in 50 mM Tris–Cl (pH 7.4) with 1% Triton X-100, 1 mM EDTA, 150 mM NaCl, and a protease inhibitor cocktail (Sigma-Aldrich, USA) using MagNa Lyser (Roche, Switzerland), and centrifuged at 14,000 rpm for 10 min at 4 °C. The protein concentration in the supernatants was determined with the Bradford method [23]. Total fractions of homogenates (50 μg per sample) were dissolved in a gel loading buffer (50 mM Tris–HCl, pH 6.8; 8% SDS, 40% glycerol and 10% 2-mercaptoethanol), heated to 95 °C and separated using 17% SDS-polyacrylamide gel electrophoresis. Next, the proteins were electroblotted onto 0.2 μm PVDF membrane (Immobilon®, Millipore, USA) for 2 hours. Then, membranes were blocked with 5% skimmed instant milk dissolved in TBS-T buffer (Tris-buffered saline, 0.1% Tween). Subsequently, membranes were incubated overnight at 4 °C with primary antibodies against MCT1 (1:1000; orb157860), MCT2 (1:1000; ARP43886_P050), and MCT4 (1:1000, ARP43796_P050). The next day, membranes were washed with TBS-T buffer and incubated with secondary antibodies (1:5000; sc-2057) conjugated with alkaline phosphatase for 1.5 h. Immunodetection was accomplished using an NBT/BCIP solution (Sigma-Aldrich, USA). Blots were photographed and counted using the ChemiDoc MP system (Bio-Rad Laboratories, Inc., CA, USA). Each sample was standardized against GAPDH (1:200; Sigma Aldrich, USA) level on each blot. Results were analyzed with Image Lab™ Software (Bio-Rad Laboratories, Inc., CA, USA).

### Data Analysis

Statistical analyses were performed using GraphPad Prism7 (MDF Co., Ltd., Japan). Data are expressed as the mean ± SEM. Data from real-time PCR and Western blot were analyzed using the Student’s *t* test. Additionally, we used one-way ANOVA to analyze results from histological staining. Differences between groups were considered statistically significant when the *p* value was < 0.05.
